# Linear functional response by two pupal *Drosophila* parasitoids foraging within single or multiple patch environments

**DOI:** 10.1371/journal.pone.0183525

**Published:** 2017-08-22

**Authors:** Gülay Kaçar, Xin-Geng Wang, Antonio Biondi, Kent M. Daane

**Affiliations:** Department of Environmental Science, Policy and Management, University of California, Berkeley, CA, United States of America; Institut Sophia Agrobiotech, FRANCE

## Abstract

Functional response describes the number of prey or hosts attacked by a predator or parasitoid as a function of prey or host density. Using three different experimental designs, we found a linear functional response by two insect parasitoids (the pteromalid *Pachycrepoideus vindemiae* and the diapriid *Trichopria drosophilae*) to their hosts (the drosophilids *Drosophila suzukii* and *D*. *melanogaster*). A linear function response is considered unusual for insect parasitoids. The first design was a ‘fixed time within patch experiment’ where individual parasitoids were exposed to a range of host densities for 24 h; the second two designs were a ‘variable time functional response’ and a ‘selective functional response’ experiments where individual parasitoids were presented with a range of host patches and allowed to freely select and explore only one patch (variable time) or forage for 24 h (selective). In all experimental designs, the number of hosts parasitized increased linearly until reaching an upper limit. Under the laboratory conditions used, the functional response of *P*. *vindemiae* was limited by its egg supply and time (host handling time) whereas *T*. *drosophilae* was limited by time only. The linear functional response by both parasitoids likely resulted from a constant attack rate and an incremental foraging strategy where the parasitoids left a poor (low density) host patch or remained in a higher quality host patch when there was successful oviposition and adequate host density.

## Introduction

Parasitoid functional response describes the number of hosts attacked by individual parasitoids as a function of host density [1], and has been both studied to assess the biological control potential of parasitoids and used as an essential component of parasitoid–host models [2–4]. Holling [1] proposed three functional response curves in response to increasing host density over a fixed time-period: a linear increase (type I) where the attack rate is constant (density independence), a negative acceleration (type II) where the attack rate gradually declines (inverse density dependence), and a sigmoidal curve (type III) where the attack rate first increases and then gradually declines (density dependence) until it reaches an upper limit due to egg availability, host handling time, or both. Host-handling time must be negligibly small for parasitoids showing a type I functional response and, in contrast, when it increases with host density or decreases because of increasing searching efficiency it will result in a type II or type III response, respectively [5–7].

The functional response models described by Holling [1] assume that both parasitoid and host populations are homogeneously distributed in space, and predict that parasitism rates are only a function of host density. When individual parasitoids are exposed to different host densities within a confined arena and over a fixed host-searching time-period, a type II functional response curve is often generated due to an increase in handling time, whereas experimental conditions that add factors such as spatial complexity within patches will often result in changes to the functional response within the fixed time-period [5, 8–11]

Studies using fixed time-periods and fixed host densities in each arena do not take into consideration that parasitoid performance may depend on its response to patchily distributed host densities [12–15]. For optimal foraging, the parasitoid should concentrate host searching in more profitable patches to maximize the reproductive success and, conversely, should leave patches with no or few hosts [16], whereas in fixed time experiments, the parasitoid may be forced to search a site repeatedly; increasing the probability that hosts are found even at low densities [3]. A design allowing a parasitoid to remain or leave different host patches would better reflect parasitoid behavior under natural conditions [17, 18]. Therefore, variable time functional response experiments, where each trial ends after a parasitoid leaves the host patch, are often used and tend to produce either type I or III curves [3, 5, 19]. A selective functional response is an alternative design, in which the host is distributed in discrete patches at different densities and exposed to individual parasitoids for a fixed time-period [20]. Using this approach, the functional response by the braconid wasp *Diachasmimorpha longicaudata* (Ashmead) shifted from type III in fixed time and within patch exposure to a bell-shape curve when the parasitoid had the chance to choose freely among different fruit fly host densities [20].

Here, we address the functional response of two solitary parasitoids, *Pachycrepoideus vindemiae* (Rondani) (Hymenoptera: Pteromalidae) and *Trichopria drosophilae* (Perkins) (Hymenoptera: Diapriidae), when attacking pupae of *Drosophila suzukii* (Matsumura) or *D*. *melanogaster* Meigen (Diptera: Drosophilidae). *D*. *suzukii* is native to East Asia but has invaded globally to include Europe, North America, and South America where it has become a major pest [21]. Although *D*. *suzukii* exploits fruit resources in the ripening stages before they become available to other *Drosophila* species, it can also infest overripe or damaged fruits concurrently with other *Drosophila* species such as *D*. *melanogaster* [22, 23]. Following the invasion of *D*. *suzukii*, several researchers in Europe and North America evaluated the potential of indigenous drosophila parasitoids to control *D*. *suzukii* in its invaded regions [24–31]. *Pachycrepoideus vindemiae* and *T*. *drosophilae* are among the few indigenous parasitoids that readily attack this invasive pest, whereas most indigenous larval parasitoids do not develop from *D*. *suzukii* because of its strong host immune resistance [22, 24, 32, 33]. Both pupal parasitoids have been found attacking *D*. *suzukii* in Europe [25, 26, 30] and North America [27–29], and are also reported from Asia and other regions [34, 35].

We explored the behavioral mechanisms underlying the functional response of *P*. *vindemiae* and *T*. *drosophilae* using three different experimental designs: fixed time and within patch, variable time, and selective functional response. To test both egg or time limitation hypotheses, we exposed individual parasitoids to different host densities for a fixed time or to a high host density at different times in the first study. To address the importance of the parasitoids’ foraging in patchy environments, in the latter two studies the host was distributed in discrete patches at different densities and exposed to individual parasitoids for variable time-periods (i.e. the experiment was ended after the parasitoid had explored one freely selected patch) or a fixed time (i.e. selective). We also compared the relative foraging efficiency between these two major parasitoids for their practical use in the biological control of the invasive *D*. *suzukii*.

## Materials and methods

### Insects

Studies were conducted under laboratory conditions (23 ± 1°C, 16L: 8D, 40–60% RH) at the University of California’s Kearney Agricultural Research and Extension Center in Parlier, California, USA. Laboratory colonies of two parasitoid species (*P*. *vindemiae* and *T*. *drosophilae*) and two host species (*D*. *suzukii* and *D*. *melanogaster*) were initiated from field collections during 2013 at this University field station and, for this reason, no specific permissions were required for these collections. *D*. *suzukii* were collected from infested cherries in the canopy and *D*. *melanogaster* were collected from rotten cherries and peaches on the ground. The parasitoids were reared from parasitized *D*. *suzukii* and *D*. *melanogaster* that had been placed in sentinel traps baited with host species pupae, as well as parasitized host pupae from field-collected fruits. To maintain vigor, field-collected insects were reintroduced into each of the colonies periodically. These were laboratory studies and the species tested were neither endangered or protected species.

Adult flies were held in Bug Dorm cages (BioQuip Products Inc., Rancho Dominguez, California, USA), while adult parasitoids were held in fine screened cages (30 × 30 × 30 cm) (Mega View Science Co. Ltd., Taichung, Taiwan); all adult insects were supplied with a 20% honey-water solution as food. Fly larvae of both species were reared on a standard cornmeal-based artificial diet in Petri dishes (1.5 cm high, 14.0 cm diameter), as described by Dalton et al. [36]. The Petri dishes were exposed to adult flies for 24 h and, after the flies had developed into 1–2 day old pupae (in 7~8 days) [37], the dishes were then exposed to adult parasitoids for 2–3 days. The parasitoid-exposed dishes were then transferred to new cages and held for the emergence of adult flies (unparasitized flies emerged in 2–3 days) or parasitoids (emerged in ~20 days).

A series of three studies were conducted, all began with 2–day old fly pupae and 4–6 day old female parasitoids that had been kept with males since emergence (i.e., assumed to be mated and with no oviposition experience and a high load of mature eggs). Host pupal age does not significantly affect the fitness of either parasitoid species [26, 28, 29].

### Fixed time and within patch functional response

To determine the fixed time and within patch functional response of *P*. *vindemiae* and *T*. *drosophilae*, host densities of 3, 6, 9, 12, 15, 18, 21 or 24 fly pupae (*D*. *suzukii* or *D*. *melanogaster*) were used, with methods identical for each host species and parasitoid-host density combination. For each replicate, fly pupae were placed on wet filter paper (to prevent desiccation) inside the center of a Petri dish (1.5 cm high × 8.5 cm diameter) and exposed to a female parasitoid for 24 h. A small streak of 50% honey-water was provisioned as food for the parasitoids. Following this 24 h exposure, the tested female parasitoids were killed and dissected under a microscope to determine the number of mature eggs retained; this was done for only tests using *D*. *suzukii*, assuming if egg limitation occurs it should be independent of host species. All exposed pupae were reared until the emergence of adult wasps or flies. Each tested combination had 30–35 replicates (a few replicates were discarded because the female wasps were found dead at the end of the exposure period). As a control, 10 replicates (each with 10 pupae) of each host species were held under the same conditions to estimate the natural mortality of fly pupae that were not exposed to parasitoids. After insect emergence ceased, all unemerged dead pupae from both treatment and control replicates were reconstituted in water for 1 day and then dissected under a microscope to determine the presence or absence of recognizable fly or parasitoid cadavers (mainly pharate adults).

The proportion of hosts parasitized at each density was estimated based on the number of emerged flies in the presence or absence of the parasitoid using the ‘Degree of Infestation (DI)’ [24, 26]:

$$DI=\frac{\left(T-di\right)}{T}$$

where T = the number of emerging flies in the absence of the parasitoid, di = the number of emerged flies in the presence of parasitoid which was determined based on the control mortality. This formula is the same as Abbott’s or Schneider-Orelli formulas that have been used to correct treatment mortality. Here, we included recognizable flies and wasps from the dissection of dead pupae to estimate the total number of emerged flies in the presence or absence of the parasitoid for a more precise estimate of DI.

Initial inspection of the data showed that *T*. *drosophilae* did not reach an upper limit at the highest density (24 pupae) tested, whereas *P*. *vindemiae* did. To estimate possible effect of egg or time limitation on the functional response, we two additional treatments with either 27 host pupae and 24 h exposure or 24 host pupae but with reduced (12 h) or extended (48 h) exposure times. The number of parasitized hosts was also estimated using the same methods as described previously and all tested females were also dissected to estimate the number of residual mature eggs (except the short exposure time treatment of 12 h). There were 30 replicates for each parasitoid species in these additional treatments. Also, we found the overall functional response patterns were similar between the tested host species and, therefore, we used only the important invasive *D*. *suzukii* as the host for the other studies.

### Variable time functional response

To determine the variable time functional response of *P*. *vindemiae* or *T*. *drosophilae*, we used five host patches (3, 6, 9, 12 and 15 *D*. *suzukii* pupae) in Petri dishes (1.5 cm high, 8.5 cm diameter), provisioned with a small streak of 50% honey-water as food for the parasitoids. The five host patches were randomly assigned to five points along the dish’s circumference, each patch was 4 cm apart and placed on a wet filter paper. A female parasitoid was released onto the center of the dish (not on any patch) and the dish was then covered. Direct behavioral observations were made to determine which patch the parasitoid first encountered (i.e. patch selection) and how long it stayed on the patch (i.e. patch residence time). After the parasitoid had freely selected and visited one patch (typically the wasp walked towards another patch), the observation was terminated and all pupae within the explored patch were dissected to determine the number of hosts parasitized. In a few cases, we discarded the parasitoid (replicate) if it did not immediately start walking towards one host patch once being released. A total of 124 and 168 females were tested successfully for *P*. *vindemiae* and *T*. *drosophilae*, respectively.

### Selective functional response

Finally, we determined the selective functional response of *P*. *vindemiae* or *T*. *drosophilae* to different host density patches. This experiment employed the same host densities and design as used for the variable time functional response experiment, but here the tested parasitoids had the possibility foraging for a fixed period of 24 h. Following the exposure period, all hosts were collected and held for the emergence of wasps or flies. As before, all dead pupae were dissected after fly or parasitoid emergence ceased. The number of hosts parasitized was also estimated using the same methods as described previously. There were 30 replicates for each parasitoid-density combination.

### Data analysis

The number of parasitized hosts as a function of host density was compared among the three experimental trials to determine the functional response for each parasitoid species. A first-order linear model with binomial errors was used to fit a logit transformation of the proportion of hosts parasitized (*p*) to host density (*N*) [7]. The model:

$$ln\;\left(\frac{p}{\left(1-p\right)}\right)=\alpha +\beta N$$

was fitted to data using the Generalized Linear Model (GLM) procedure. A type I response is characterized by no dependence of *p* on *N*, a type II response by a negative dependence, and a type III response by a positive dependence. It is important that the data set be restricted to the host density range for which the numbers of parasitized hosts fall below the upper limit of the functional response to host density [7]. For this reason, we used data with 3–18 hosts for *P*. *vindemiae* and 3–24 hosts for *T*. *drosophilae* (see results) to avoid the upper limit of the functional response to host density.

Data for the fixed time functional response were further analyzed to compare the effects of parasitoid species, host density, and host species on the number of hosts parasitized using GLM with normal distribution and an identity link function. Data for each parasitoid-host combination were separately analyzed to compare the mean number of hosts parasitized among different host densities using one-way ANOVA, with mean separation using Tukey’s HSD. A linear model was then used to fit the relationship between the number of hosts parasitized and host density when the number of parasitized hosts falls below the upper limit of the functional response. The effect of different exposure times on the number of hosts parasitized or the number of retained mature eggs was analyzed for each parasitoid species separately using one-way ANOVA and Tukey’s HSD.

For the variable time functional response, the frequency of first encounter with different host density patches was analyzed using the *χ*^*2*^ Goodness of Fit test for each parasitoid species. The effects of host density and parasitoid species on the patch residence time, number of hosts parasitized, or attack rate (number of hosts parasitized per unit of time) were analyzed using GLM with Normal distribution and an identity link function (patch time and number of hosts parasitized) or Poisson distribution and a log-link function (attack rate). A linear model was used to fit the relationship between the number of hosts parasitized and host density and between the patch residence time and host density for each parasitoid species. Data for the selective functional response were further analyzed to determine the effects of host density and parasitoid species on the number of hosts attacked using GLM with Normal distribution and an identity link function. A linear model was used to fit the relationship between the number of hosts parasitized and host density for each parasitoid species. All data analyses were performed using JMP Pro ver13 (SAS 2013, Cary, NC).

## Results

### Fixed time functional response

**Fig 1:**
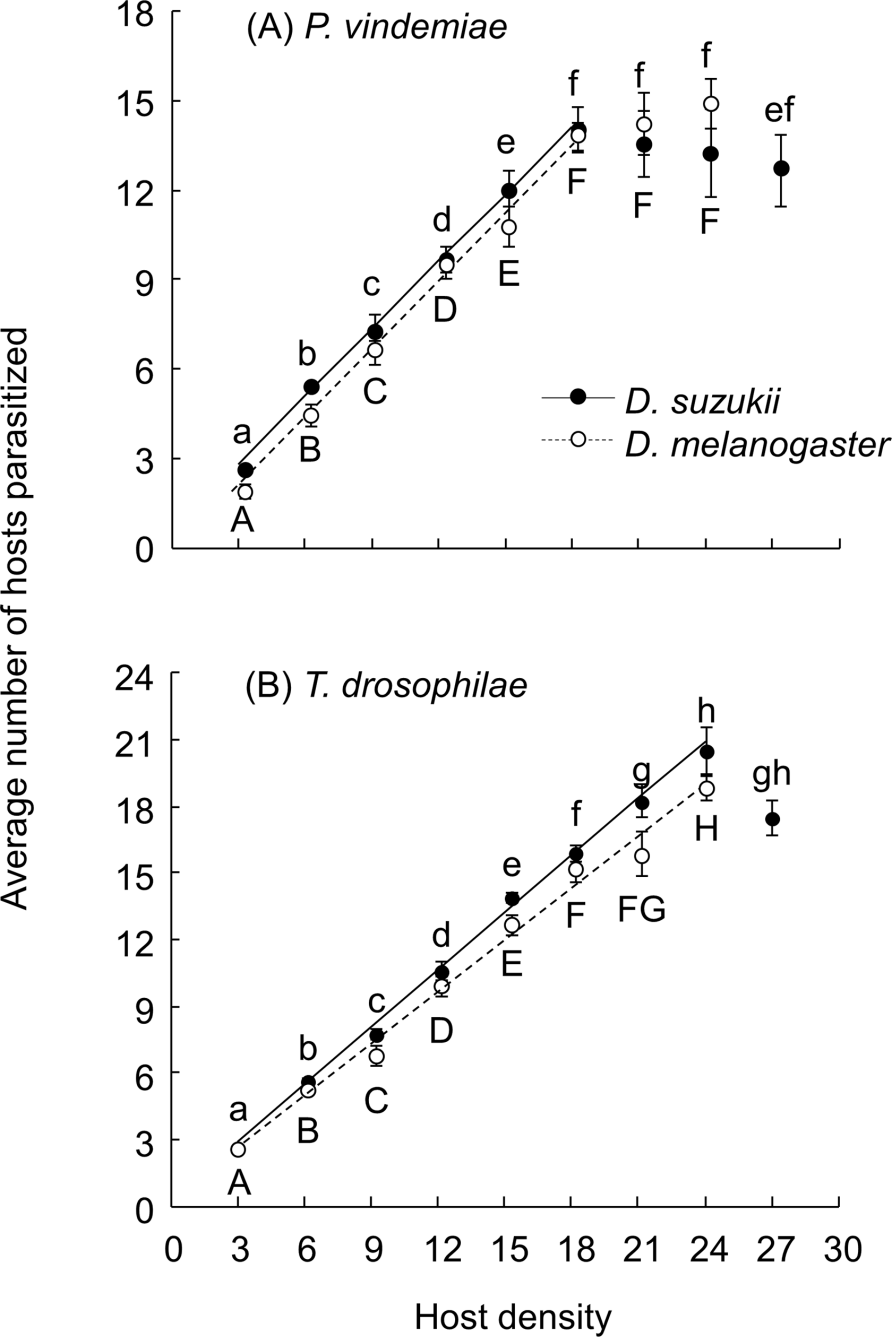
Fixed time functional response of parasitoids to different host patch densities of their drosophilid hosts. Data for the functional response of (A) *P*. *vindemiae* and (B) *T*. *drosophilae* to the host pupal density of *D*. *suzukii* or *D*. *melanogaster* within the host density range from 3 to 18 (*P*. *vindemiae*) and 3 to 24 (*T*. *drosophilae*) were fitted to a linear model and are: *y* = 0.594 + 0.754 *x*, *R*^2^ = 0.998; *y* = – 0.293 + 0.777 *x*, *R*^2^ = 0.992; *y* = 0.311 + 0.857 *x*, *R*^2^ = 0.997; *y* = 0.431 + 0.774 *x*, *R*^2^ = 0.989 for *P*. *vindemiae* on *D*. *suzukii* and *D*. *melanogaster* and for *T*. *drosophilae* on *D*. *suzukii* and *D*. *melanogaster*, respectively. Data (mean ± SE) were analyzed for each host-parasitoid combination separately and different letters indicate significant difference among different host densities of *D*. *suzukii* (lower case) or *D*. *melanogaster* (upper case) (ANOVA and Tukey’s HSD, *P* < 0.05).

The number of hosts parasitized increased with increasing host density to upper limits of 18 hosts for *P*. *vindemiae* and 24 hosts for *T*. *drosophilae* during the 24 h exposure period. The functional response curves generated were similar between the two drosophilid species (Fig 1). Using the data sets that were restricted to 3–18 hosts for *P*. *vindemiae* and 3–24 hosts for *T*. *drosophilae* the functional response by both parasitoids on both host species are type I (Table 1). The ‘area searched’ was the slope of the linear curve (Fig 1) and was 0.754 (0.707–0.801, 95% CI) and 0.777 (0.681–0.873, 95% CI) for *P*. *vindemiae* on *D*. *suzukii* and *D*. *melanogaster*, respectively, and 0.857 (0.808–0.905, 95% CI) and 0.774 (0.696–0.852, 95% CI) for *T*. *drosophilae* on *D*. *suzukii* and *D*. *melanogaster*, respectively.

**Table 1:** General linear models used to test for host density dependence in the functional response of *Pachycrepoideus vindemiae* and *Trichopria drosophilae* using a binomial error distribution and logit transformation of the proportion of hosts parasitized.

Functional response experiment	Parasitoid species	Host species	Host density range	*χ*^2^	df	*P*
**Fixed time**	*P*. *vindemiae*	*D*. *suzukii*	3–18	0.063	1	0.832
		*D*. *melanogaster*	3–18	0.041	1	0.854
	*T*. *drosophilae*	*D*. *suzukii*	3–24	0.007	1	0.931
		*D*. *melanogaster*	3–24	0.027	1	0.871
**Variable time**	*P*. *vindemiae*	*D*. *suzukii*	3–15	0.051	1	0.822
	*T*. *drosophilae*	*D*. *suzukii*	3–15	0.046	1	0.830
**Selective**	*P*. *vindemiae*	*D*. *suzukii*	3–15	0.001	1	0.981
	*T*. *drosophilae*	*D*. *suzukii*	3–15	0.033	1	0.855

**Fig 2:**
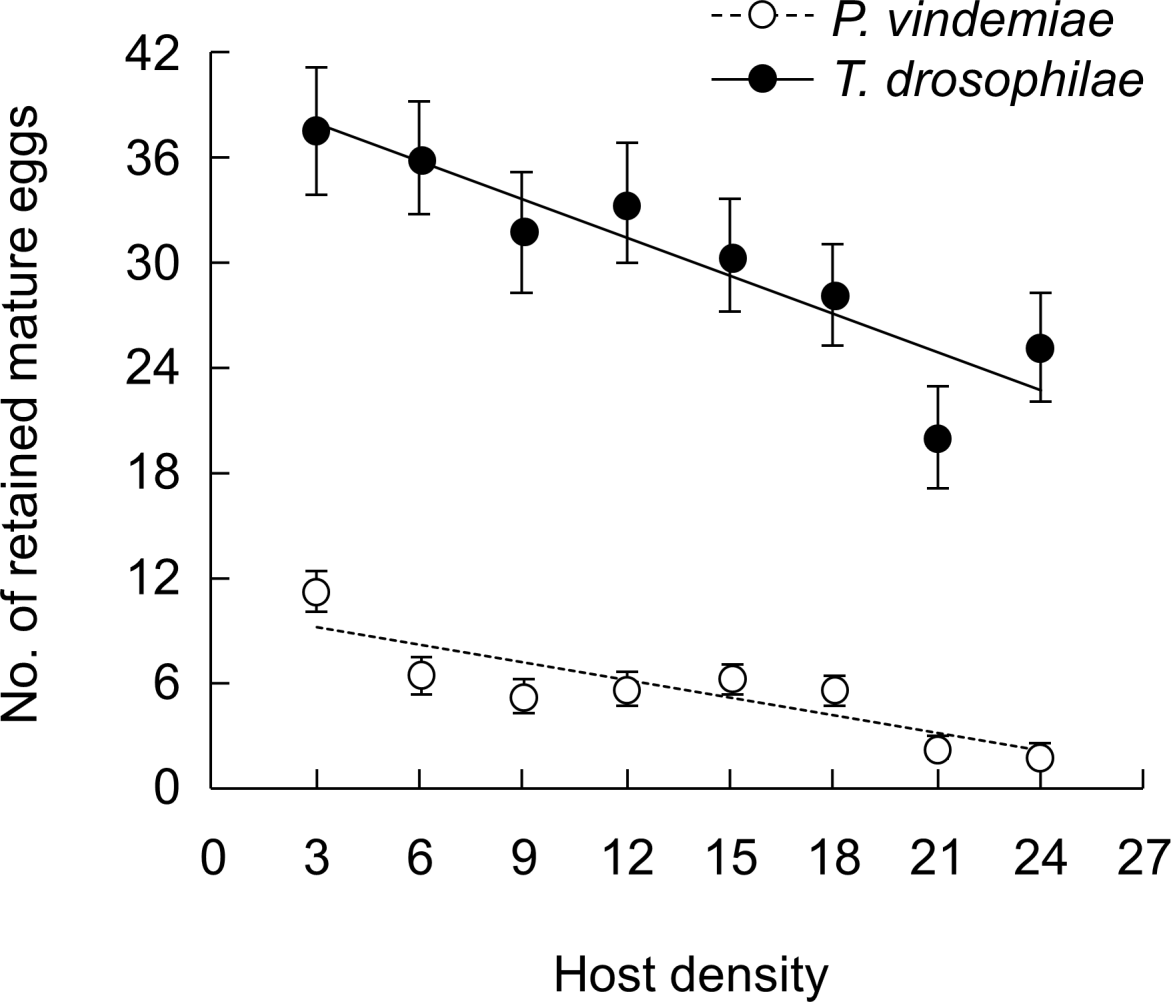
Retained mature parasitoid eggs after exposure to drosophilid hosts. The number of retained mature eggs of *P*. *vindemiae* or *T*. *drosophilae* after the exposure to different densities of *D*. *suzukii* pupae for 24 h in the fixed time functional response experiment. The data (mean ± SE) were fitted to a linear model for both *P*. *vindemiae* (*y* = 10.163–0.338 *x*, *R*^2^ = 0.741) and *T*. *drosophilae* (*y* = 40.067–0.718 *x*, *R*^2^ = 0.830).

**Fig 3:**
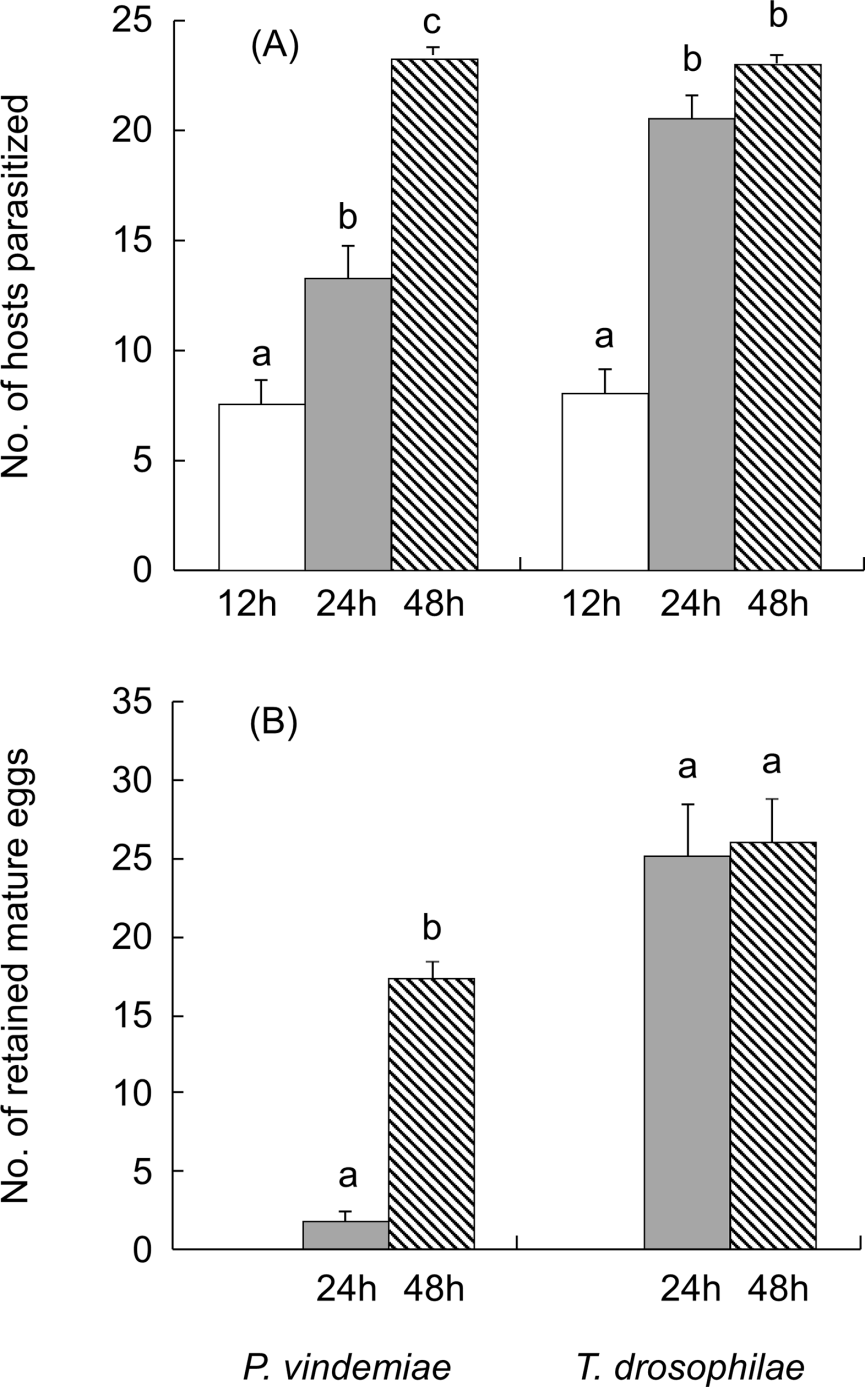
Time or egg limitation by the parasitoids. (A) The number of hosts parasitized by *P*. *vindemiae* and *T*. *drosophilae* after a female parasitoid was exposed to 24 *D*. *suzukii* pupae for 12, 24 or 48 h, respectively, and (B) the number of retained mature eggs by *P*. *vindemiae* and *T*. *drosophilae* after the 24 and 48h exposure. Values are mean ± SE and different letters above the bars indicate significant difference among the different exposure times (ANOVA and Tukey’s HSD, *P* < 0.05).

GLM analyses showed the number of hosts parasitized (within the density range of 3–24) was influenced by host density (*χ*^2^ = 2285.6, df = 1, *P* < 0.001), host species (*χ*^2^ = 165.3, df = 1, *P* < 0.001) and parasitoid species (*χ*^2^ = 10.2, *P* = 0.001). Overall, *P*. *vindemiae* parasitized fewer hosts than *T*. *drosophilae*, and more *D*. *suzukii* were parasitized than *D*. *melanogaster* by each parasitoid species (Fig 1). The number of retained eggs by both parasitoids decreased with increasing number of hosts parasitized, and *P*. *vindemiae* retained fewer mature eggs than *T*. *drosophilae* (Fig 2). Further experiments with the reduced (12 h) or extended (48 h) exposure time at the density of 24 hosts showed that the number of parasitized hosts increased with the exposure time by *P*. *vindemiae* but there was no difference between the 24 and 48 h for *T*. *drosophilae* (Fig 3A). Therefore, *P*. *vindemiae* appears to have depleted its egg supply after the 24 h exposure, but developed more eggs at 48 h, whereas *T*. *drosophilae* held more than 25 mature eggs at both time periods (Fig 3B), suggesting that *P*. *vindemiae* likely had suffered from egg supply while *T*. *drosophilae* was not yet egg-limited or time-limited when the functional response curve approached to an asymptote at the high host densities for a fixed exposure time.

### Variable time functional response

The frequency distributions of the first encounter with the five different host density patches (3, 6, 9, 12 and 15 pupae) were 17, 20, 21, 29 and 37 in *P*. *vindemiae* and 17, 24, 38, 46 and 43 in *T*. *drosophilae*, respectively. Thus, wasps first located and moved onto the higher density host patches (*P*. *vindemiae*: *χ*^2^_0.05, 4_ = 10.7, *P <* 0.05 and *T*. *drosophilae*: *χ*^2^_0.05, 4_ = 18.7, *P <* 0.01).

**Fig 4:**
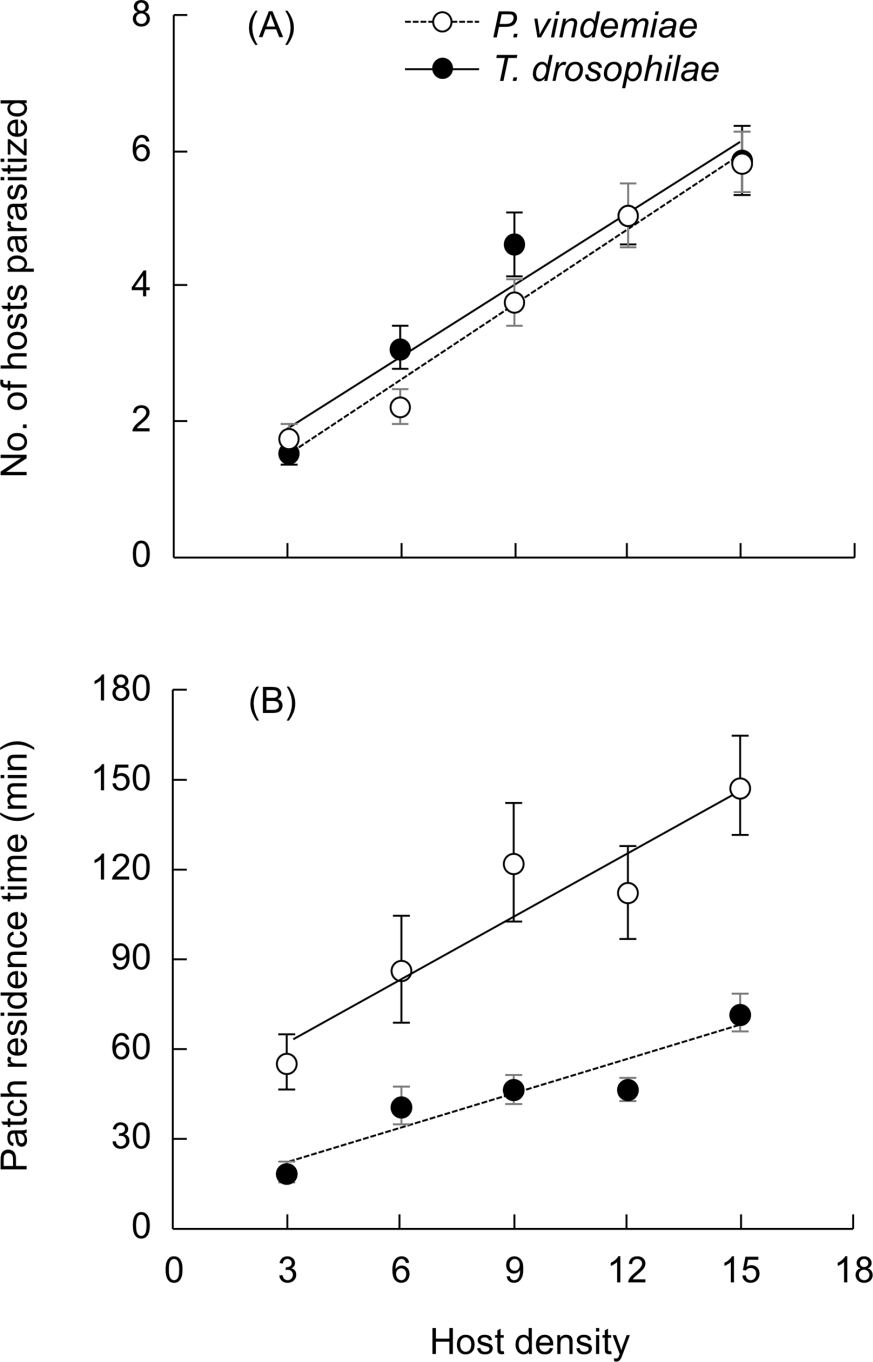
The variable time functional response of *P*. *vindemiae* and *T*. *drosophilae* to the host pupal densities of *D*. *suzukii*. Five different host density patches were presented to individual female parasitoids and the experiment was ended after the parasitoid had explored one patch: (A) number of hosts parasitized, (B) patch residence time. The data (mean ± SE) were fitted to a linear model for both patch residence time (*P*. *vindemiae*: *y* = 41.818 + 7.033 *x*, *R*^2^ = 0.888; *T*. *drosophilae*: *y* = 11.550 +3.728 *x*, *R*^2^ = 0.869) and the number of hosts parasitized (*P*. *vindemiae*: *y* = 0.848 + 0.354 *x*, *R*^2^ = 0.951; *T*. *drosophilae*: *y* = 0.439 + 0.366 *x*, *R*^2^ = 0.898).

**Fig 5:**
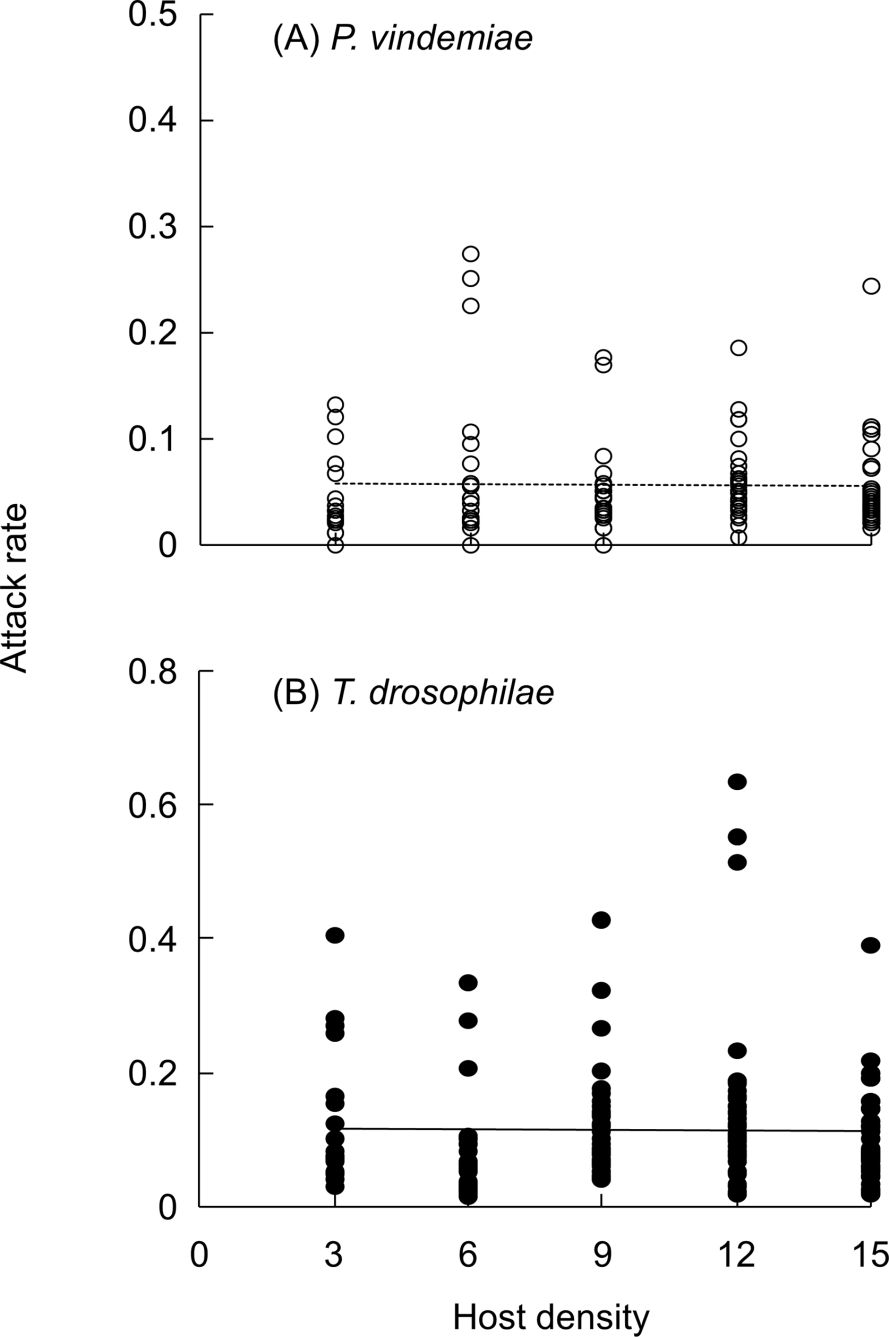
Constant attack rate (number of hosts parasitized per unit time) by parasitoids. The attack rate by (A) *P*. *vindemiae* and (B) *T*. *drosophilae*, showing the constant attack rate across the different host density patches in the variable time functional response.

GLM analyses indicate patch residence time was affected by host density (*χ*^2^ = 34.1, *P* < 0.001) and parasitoid species (*χ*^2^ = 68.2, *P* < 0.001), whereas the number of hosts parasitized was affected by host density (*χ*^2^ = 91.1, *P* < 0.001) but not parasitoid species (*χ*^2^ = 0.653, *P* = 0.042). The number of hosts parasitized increased linearly with host density (Fig 4A). Logistic regression analyses of the proportion of hosts parasitized and the host density suggest the type I response (Table 1). The patch time also increased linearly with host density but *P*. *vindemiae* stayed longer than *T*. *drosophilae* on each patch it visited (Fig 4B). Consequently, the attack rate was independent of host density (*χ*^2^_0.05, 4_ = 0.028, *P* = 0.866) for each parasitoid species, although it was lower with *P*. *vindemiae* (Fig 5A) than *T*. *drosophilae* (Fig 5B) (*χ*^2^_0.05, 4_ = 33.1, *P <* 0.001). The mean attack rate per host per minute was 0.057 ± 0.007 for *P*. *vindemiae* and 0.115 ± 0.006 for *T*. *drosophilae*. Dissection of all parasitized hosts found that self-superparasitism was negligible (< 1%) by both parasitoid species when the female parasitoid could freely select and leave the patch in this variable time experiment.

### Selective functional response

**Fig 6:**
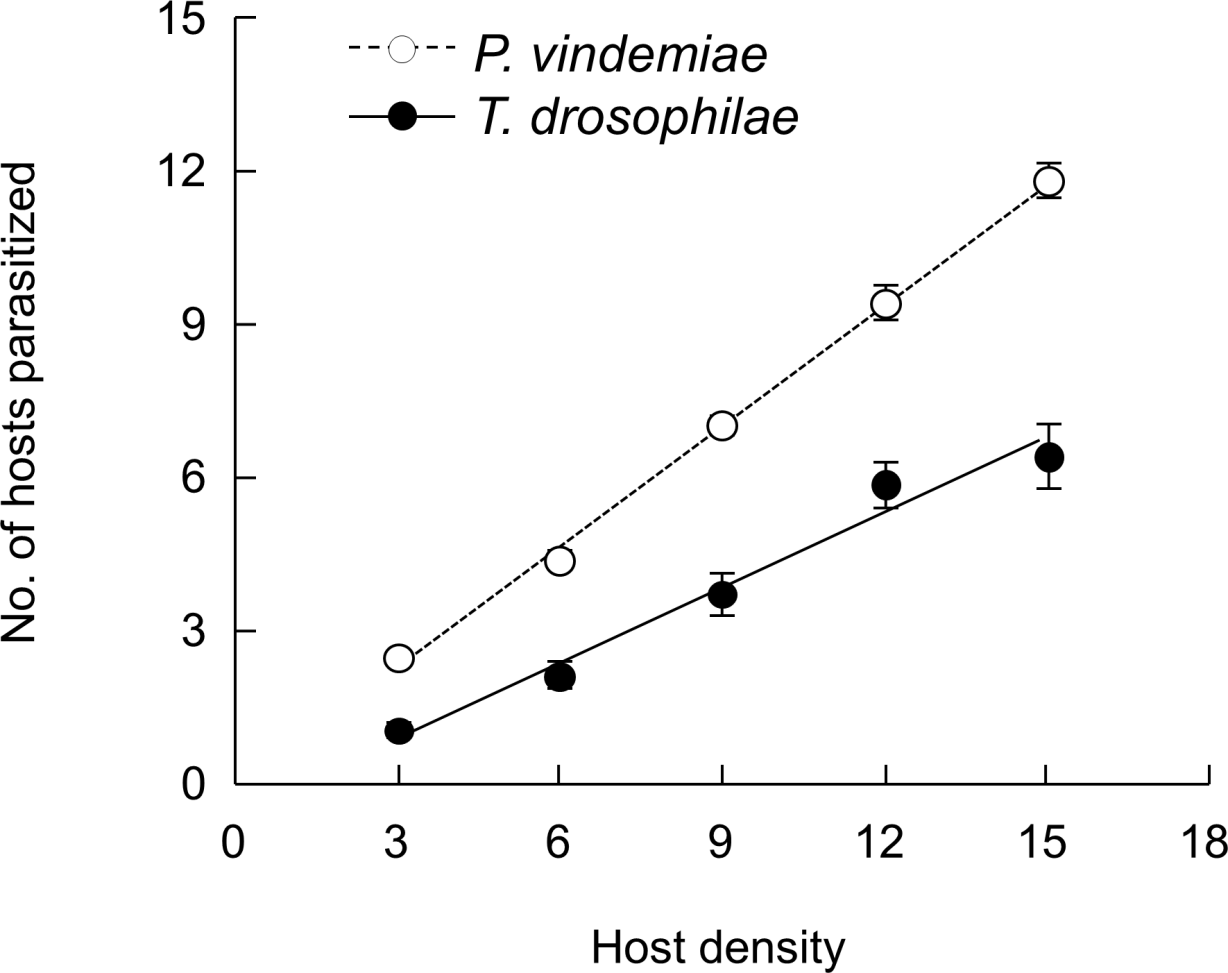
The selective functional response of *P*. *vindemiae* and *T*. *drosophilae* to the host pupal densities of *D*. *suzukii*. Five different host density patches were simultaneously presented to individual female parasitoids for 24 h. The data (mean ± SE) were fitted to a linear model for both *P*. *vindemiae* (*y* = – 0.490 + 0.480 *x*, *R*^2^ = 0.441) and *T*. *drosophilae* (*y* = – 0.076 + 0.787 *x*, *R*^2^ = 0.861).

GLM analyses indicate that the number of hosts parasitized was affected by both the host density (*χ*^2^ = 141.8, *P* < 0.001) and the parasitoid species (*χ*^2^ = 441.5, *P* < 0.001) (Fig 6). Logistic regression analyses of the proportion of hosts parasitized and the host density suggest the type I response (Table 1). Thus, the number of hosts parasitized increased linearly with host density, but more hosts were parasitized by *T*. *drosophilae* than *P*. *vindemiae* (Fig 6).

## Discussion

We demonstrated the linear (type I) functional response by *P*. *vindemiae* and *T*. *drosophilae* foraging in a single host patch for a fixed time or in host patches of different densities for either a variable or fixed time. Examples of type I function responses are somewhat rare [38], but have been reported in some parasitoid species, including trichogrammid and mymarid egg parasitoids [7, 39, 40], aphelinid parasitoids of whiteflies [8, 41], and braconid parasitoids of lepidopterans [42, 43]. Theoretically, a ‘true’ type I functional response is possible when the handling time is negligibly small [3, 5, 19], or a predator can search and handle different prey simultaneously such as filter feeders that take food at a constant rate and stop abruptly once saturated [6, 44]. However, handling times are often negligibly small in insect parasitoids, and obviously, parasitoids are not able to parasitize one host while simultaneously searching for or handling another.

One aspect of our study was the use of three different experimental designs and our expectation that one of these trials would have resulted in the more common type II curve. The functional responses of both *P*. *vindemiae* and *T*. *drosophilae*, as measured by both attack rate and search strategy, to changing host density still produced the more unusual type I response. Our studies were conducted in simple laboratory arenas and, under more natural settings, factors such as host distribution, spatial complicity and interference among parasitoids could affect a parasitoid attack rate [7, 17]. For example, Ives et al. [9] measured the attack rate by the braconid *Aphidius ervi* Haliday on the aphid *Acyrthosiphon pisum* (Harris) and found spatial variability such that a decreasing attack rate at low host density transformed a strong type II functional response into one approaching type I. When foraging for patchy resources, the change of a parasitoid’s searching strategy may depend on its ability to assess patch quality. Waage [15] and Driessen et al. [14] proposed different behavioral responses that assume parasitoids should leave or remain in a host patch depending on the number of successful ovipositions over time. Our results agree with Waage's model prediction as both patch residence time and oviposition success increased with host density in our multiple patch experiment. Both *P*. *vindemiae* and *T*. *drosophilae* may enter a patch with imperfect information about patch quality, but leave that patch if there are few hosts or stay longer if there are more hosts, as observed in our variable time experiment. Naturally, if the time needed for a parasitoid to attack each host is constant in each patch, it will stay longer in those patches where there are more hosts because it will encounter more hosts per search time and the total patch residence time will then increase linearly with the number of hosts present [16, 19, 45].

The number of hosts parasitized by *P*. *vindemiae* and *T*. *drosophilae* in high host density patches could have been limited by the time-period imposed in our trials–ending the trial before the parasitoids could deposit all their mature eggs, or by the parasitoid’s available egg load–if the parasitoid used all her eggs and there were still unparasitized hosts. Both *P*. *vindemiae* and *T*. *drosophilae* are mostly synovigenic and emerge with a partial complement of their lifetime egg load [28, 34]. However, *P*. *vindemiae*, like most ectoparasitoids, typically produces and holds only a few large eggs (< 20) at a time [12], whereas *T*. *drosophilae* is an endoparasitoid and produces and holds a higher number of mature eggs (40–60) [28]. Our fixed time trials showed this difference in parasitoid egg availability, *P*. *vindemiae* depleted almost all eggs after 1 d foraging in the high host density patches whereas *T*. *drosophilae* deposited only about 50% of the available mature eggs. At 2 d, *P*. *vindemiae* had matured more eggs, suggesting this parasitoid will deposit all available eggs when hosts are present, exhibiting a behavioral plasticity to adjust egg production in response to variations in host supply. This suggests that when foraging in high host density patches, daily rates of *P*. *vindemiae* parasitism might be limited from both egg supply and foraging time, whereas *T*. *drosophilae* might be, relatively, limited by time only.

In our laboratory trials, *T*. *drosophilae* had a higher overall attack rate than *P*. *vindemiae* against *D*. *suzukii*, although many other factors affect parasitoid efficiency [28, 29] and *P*. *vindemiae* is often more commonly collected than *T*. *drosophilae* in field studies. Under similar conditions, juvenile mortality on *D*. *suzukii* was similar between the two parasitoids (~15% mortality) [28, 30]. Although female *T*. *drosophilae* have a higher load of mature eggs than female *P*. *vindemiae*, offspring of *P*. *vindemiae* were female-biased and consequently the intrinsic rate of increase of *T*. *drosophilae* (0.124) was similar to that of *P*. *vindemiae* (0.139) on *D*. *suzukii* under similar conditions [28, 30]. In fact, biological control success does not appear to be directly or exclusively related to the functional response curve [38]. We must point out that the current experiments were conducted in the laboratory in small arenas and had used host densities that may not reflect field populations. Many frugivorous *Drosophila* larvae, such as *D*. *melanogaster*, breed on fallen or decaying fruits where there is often found a clumped pattern in terms of host availability, with fruits containing either high or low numbers of hosts. In contrast, *D*. *suzukii* attacks fresh fruit and often deposits only one or a few eggs in each fruit, and its eggs may be more discretely distributed across fruits in the field [46]. In this situation, the parasitoids would have to search greater areas and use longer time to locate similar numbers of *D*. *suzukii*. Thus, laboratory studies may not accurately reflect field results because of differences in host density and distribution [8, 42]. Moreover, field conditions also provide changes in host refuge [47], multiple prey or host species [48], and/or pesticide exposure [49] that can influence parasitoid behavior and functional response. We chose the simplest scenario, that could help us to identify some behavioral mechanisms underlying the linear host density response. To further understand the behavioral and ecological mechanisms, we must document how and to what extent host availability and distribution pattern may influence a parasitoid’s searching strategy and the resultant pattern of host density dependence under more realistic natural conditions.

## Supporting information

S1 File This is raw data (in microsoft excel) supporting information file. (XLSX)

## Abbreviations

h: hour
L: light
D: dark
RH: relative humidity
ANOVA: analysis of variance
GLM: generalized linear model
